# Preclinical investigations of the efficacy of the glutaminase inhibitor CB-839 alone and in combinations in chronic lymphocytic leukemia

**DOI:** 10.3389/fonc.2023.1161254

**Published:** 2023-05-09

**Authors:** Natalia Timofeeva, Mary L. Ayres, Natalia Baran, Janice M. Santiago-O’Farrill, Gamze Bildik, Zhen Lu, Marina Konopleva, Varsha Gandhi

**Affiliations:** ^1^ Department of Experimental Therapeutics, The University of Texas MD Anderson Cancer Center, Houston, TX, United States; ^2^ Department of Leukemia, The University of Texas MD Anderson Cancer Center, Houston, TX, United States

**Keywords:** CLL - chronic lymphoblastic leukemia, BTK - Bruton’s tyrosine kinase, Bcl-2 inhibitor, Mcl-1 inhibitor, ibrutinib, venetoclax, glutamine (Gln) metabolism

## Abstract

**Introduction:**

Chronic lymphocytic leukemia (CLL) cells are metabolically flexible and adapt to modern anticancer treatments. Bruton tyrosine kinase (BTK) and B-cell lymphoma-2 (BCL-2) inhibitors have been widely used to treat CLL, but CLL cells become resistant to these treatments over time. CB-839 is a small-molecule glutaminase-1 (GLS-1) inhibitor that impairs glutamine use, disrupts downstream energy metabolism, and impedes the elimination of reactive oxygen species.

**Methods:**

To investigate the *in vitro* effects of CB-839 on CLL cells, we tested CB-839 alone and in combination with ibrutinib, venetoclax, or AZD-5991 on the HG-3 and MEC-1 CLL cell lines and on primary CLL lymphocytes.

**Results:**

We found that CB-839 caused dose-dependent decreases in GLS-1 activity and glutathione synthesis. CB-839–treated cells also showed increased mitochondrial superoxide metabolism and impaired energy metabolism, which were reflected in decreases in the oxygen consumption rate and depletion of the adenosine triphosphate pool and led to the inhibition of cell proliferation. In the cell lines, CB-839 combined with venetoclax or AZD-5991, but not with ibrutinib, demonstrated synergism with an increased apoptosis rate and cell proliferation inhibition. In the primary lymphocytes, no significant effects of CB-839 alone or in combination with venetoclax, ibrutinib, or AZD-5991 were observed.

**Discussion:**

Our findings suggest that CB-839 has limited efficacy in CLL treatment and shows limited synergy in combination with widely used CLL drugs.

## 1 Introduction

Metabolic reprogramming plays a pivotal role in cancer development and patient survival. Compared to other B-cell malignancies, chronic lymphocytic leukemia (CLL) is not highly active metabolically (1); however, it develops metabolic modifications that underlie its progression and resistance to drugs (2–4). Some of these modifications affect oxidative phosphorylation (OXPHOS) and help cancer cells use alternatives to glucose substrates to produce adenosine triphosphate (ATP) (5). ATP, an end product of OXPHOS, provides fuel to meet CLL cells’ high energy requirements. It has been shown that pharmacological depletion of ATP inhibits RNA synthesis and leads to the apoptosis of CLL cells (6).

OXPHOS depends on the activity of the tricarboxylic acid (TCA) cycle, which produces energy precursors for the electron transport chain. Acetyl-CoA produced from glucose is the most well-known substrate for the TCA cycle. However, glutamine is a major driver for OXPHOS in cancer cells, and glutamine limitation, rather than glucose contributes to lower oxygen uptake and mediates the apoptosis of cancer cells (7, 8). The initial step required for the glucose-independent fueling of OXPHOS is the conversion of glutamine to glutamate. Subsequently, glutamate provides a substrate for the synthesis of α-ketoglutarate, the key metabolite of the TCA cycle (9).

The rate-limiting mitochondrial enzyme in glutamine metabolism is glutaminase, which catalyzes the conversion of glutamine into glutamate and ammonia. Glutaminase has 2 isoforms: kidney-type glutaminase-1 (GLS-1) and liver-type glutaminase-2. GLS-1, in turn, has 2 alternative splice variants: glutaminase C (GAC) and kidney glutaminase (KGA). Glutaminase C has higher catalytic activity than kidney glutaminase and is commonly upregulated in leukemia cells (10, 11). It has been shown that knockdown of the GLS-1 gene in acute myeloid leukemia (AML) cell lines disrupted glutamine-driven OXPHOS, resulting in reduced cell proliferation and the induction of apoptosis (10). This finding indicated that drugs that alter glutamine use may be useful in CLL therapy.

CLL cells are highly dependent on the B-cell receptor pathway, which provides signaling for cell development and maturation. The endpoint of B-cell receptor stimulation is the activation of the NF-κB and MAP kinase pathways, which results in the proliferation, migration, and survival of CLL cells. Bruton tyrosine kinase (BTK) plays a critical role in signal transduction via the B-cell–receptor signaling cascade. Thus, it became an effective target for covalent BTK inhibitors such as ibrutinib (12).

The most frequent cytogenetic mutation in CLL is the 13q deletion (del[13q]), found in around 50% of CLL cases (13, 14). In del[13q]CLL cells, the microRNA (miR) cluster miR-15a/miR-16-1 is deleted, leading to the loss of its tumor suppression function and overexpression of the antiapoptotic proteins B-cell lymphoma-2 (BCL-2) and myeloid cell leukemia-1 (MCL-1). Dysregulated BCL-2 expression contributes to leukemic cell survival and accumulation, while the MCL-1 protein exerts a protective effect on CLL cells, inhibiting apoptosis (15, 16). Thus, the BCL-2 inhibitor venetoclax and the MCL-1 inhibitor AZD-5991 have potent targets for CLL treatment.

One of the biggest challenges in treating CLL is the development of resistance to BTK and BCL-2 inhibitors. Continuous treatment with anti-BTK and anti-BCL-2 therapies is typically required in the management of CLL, and the success of these therapies is heavily dependent on the emergence of drug resistance over time. Metabolic reprogramming, which leads to effects such as a higher oxygen consumption rate (OCR) and altered glutamine and glucose metabolism in cells, contributes to the cells’ resistance to venetoclax and ibrutinib (4, 17). An examination of the transcriptomic landscape of ibrutinib-resistant mantle cell lymphoma cells revealed enhanced reliance on OXPHOS and glutaminolysis (18). Furthermore, ibrutinib-resistant CLL cells demonstrate increased glutamine uptake, and ibrutinib causes glutamate depletion and promotes α-ketoglutarate synthesis (19). Thus, targeting dysregulated glutaminolysis may be an option to overcome drug resistance. It has been reported that the inhibition of GLS-1 sensitizes AML cells to priming with venetoclax and activates mitochondrial apoptosis (10).

CB-839 (Telaglenastat), a potent, reversible, and selective small-molecule inhibitor that targets GLS-1 and has a half-maximal inhibitory concentration (IC_50_) of 24 nM for recombinant human glutaminase C, has been investigated in solid tumors and in AML, diffuse large B-cell lymphoma, and multiple myeloma cells alone or in combination with other anticancer drugs (10, 11, 20–26). The inhibition of GLS-1 *in vitro* decreased the concentration of downstream glutaminase metabolites (glutamate, α-ketoglutarate, aspartate, fumarate, and malate) and induced cell death (10, 11).

We hypothesize, that dysregulation of glutaminolysis using CB-839 may overcome drug resistance in CLL cells and be an effective treatment strategy when used alone or in combination with agents that inhibit pathways responsible for survival, proliferation, and migration of CLL lymphocytes. These pathways include BCL-2 (venetoclax), BTK and BCR (ibrutinib) and MCL-1 (AZD-5991). In this study, we investigated the *in vitro* effects of CB-839, alone or in combination therapy, in the HG-3 and MEC-1 CLL cell lines and on primary CLL lymphocytes.

## 2 Materials and methods

### 2.1 Cell lines

We used MEC-1 cells from a CD5^low^/^−^ CLL cell line established from a patient with CLL in transformation to B-cell prolymphocytic leukemia (generously provided by Dr. Sabrina Bertilaccio) (27). These cells have a complex karyotype that includes del(17p). The HG-3 wild-type cell line was a kind gift from the laboratory of Dr. Deepa Sampath (at The University of Texas MD Anderson Cancer Center). Cells were cultured in Roswell Park Memorial Institute (RPMI)-1640 medium (Corning) and supplemented with 10% fetal bovine serum and penicillin-streptomycin (100 U/μg/mL; Invitrogen). Cell numbers were determined over time using a Beckman Z2 Cell Counter (Coulter). Cell lines were authenticated using a short tandem repeats assay and were tested frequently for mycoplasma contamination.

### 2.2 Patient samples

Peripheral blood was obtained from 30 treatment-naïve patients with CLL who provided written informed consent as part of a protocol approved by the Institutional Review Board of MD Anderson Cancer Center and in accordance with the Declaration of Helsinki. Patient characteristics are provided in **Supplemental Table 1**. Blood samples were diluted with phosphate-buffered saline, and mononuclear cells were isolated using the Ficoll-Hypaque density gradient centrifugation method. Isolated mononuclear cells were considered as primary CLL lymphocytes because all patients had absolute lymphocytosis (median white blood cells count 48.75 × 10^9^/L) associated with CLL. Cells were seeded at a density of 1 × 10^6^ cells/mL and were cultured for 24 hours (n = 11) or 72 hours (n = 19) in RPMI-1640 medium supplemented with 10% human serum (Sigma-Aldrich) and penicillin-streptomycin (100 U/μg/mL). To maintain the viability of the primary CLL cells during their 72-hour culturing, the cells were supplemented with CD40 ligand (20 ng/mL; Gibco) and recombinant human interleukin-4 (10 ng/mL; Gibco). Cell numbers were determined over time using a Beckman Z2 Cell Counter.

### 2.3 Drugs

CB-839 (0.1-100 μM; Selleckchem), L-buthionine-(S,R)-sulfoximine (BSO; 1-500 μM; Selleckchem), ibrutinib (1-20 μM; Selleckchem), venetoclax (0.01-10 μM; XcessBio), and AZD-5991 (0.25-10 μM; ChemieTek) were used in various concentrations and combinations for the treatment. Rapamycin (Thermo Fisher), bafilomycin A1 (Sigma-Aldrich), and chloroquine (Sigma-Aldrich) were used for the autophagy assays. For all drugs, except BSO, stock solutions were made in dimethyl sulfoxide (DMSO; Sigma-Aldrich), and BSO was reconstituted in molecular-grade water (Corning). DMSO only in matching concentrations (0.1-1%) was used as the vehicle control.

### 2.4 Metabolic measurements of glutamine, glutamate, and glutathione

For the luminescent assays, the MEC-1 and HG-3 cells were seeded in triplicate with CB-839 (0.1-100 μM). After 72 hours of incubation, cells were counted and 50,000 cells per each condition were taken for the luminescent assays. To determine the intracellular glutamate concentrations, a Glutamine/Glutamate-Glo Assay (Promega) was used following the manufacturer’s instructions. The reduced glutathione levels were measured using a GSH-Glo Assay (Promega) according to the manufacturer’s instructions. Luminescence was measured using a BioTek Synergy HT microplate reader. Metabolite concentrations were calculated using a standard curve method.

### 2.5 Reactive oxygen species and superoxide measurements

For the cellular reactive oxygen species (ROS) measurements, treated cells were stained with 20 μM 2′,7′–dichlorofluorescin diacetate (DCFDA; Abcam) for 30 minutes at 37°C in a humidified atmosphere containing 5% CO_2_ and were treated for 4 hours with the drugs in serum-free media protected from light. For the mitochondrial superoxide (SOX) measurements, treated cells were stained with 5 μM of selective MitoSOX reagent (Invitrogen) and incubated in serum-free media for 10 minutes at 37°C in a humidified atmosphere containing 5% CO_2_. After staining, all cells were washed and resuspended in phosphate-buffered saline and were immediately analyzed using flow cytometry. The results were processed using FlowJo software (FlowJo, LLC). The gating strategy is described in **Supplemental Figure 1**. A geometric mean ± the standard deviation was used for the evaluation of ROS production and compared among different treatment conditions.

### 2.6 Cell viability assays

For the apoptosis assay, an annexin V-FITC kit (BD Pharmingen) and propidium iodide (PI; Sigma-Aldrich) staining were used. Cells were resuspended in annexin V binding buffer (BD Pharmingen) for staining and were analyzed using flow cytometry after 15 minutes of incubation at FL-1 and FL-3 channels. Cells that were negative for both stains were considered viable.

For the cell proliferation assay, the MTS method was used (CellTiter96 proliferation assay; Promega). Cells were seeded at a density of 10,000 cells/well in 100 μL of complete media in triplicate and were incubated for 72 hours with 10 nM, 100 nM, 1 μM, 10 μM, and 100 μM of CB-839. DMSO was used as a control vehicle. After the treatment, 20 μL of reagent was added to a 96-well plate containing the cell suspensions, and the plate was incubated for 4 hours. Color reactions were quantified using a BioTek Synergy HT microplate reader at 490 nm.

### 2.7 Thymidine and uridine incorporation assays

MEC-1 and HG-3 cells were seeded at a density of 1 × 10^6^ cells/mL in 3 mL of complete RPMI-1640 medium in a T25 flask. CB-839 was added to the final concentrations of 10 nM, 100nM, 1 μM, 10 μM, and 100 μM in triplicate, and the cells were incubated for 72 hours. DMSO was used as a control vehicle. After incubation, 3μCi of [methyl-^3^H] thymidine (Moravek Biochemicals; MT-6036) or 3 μCi of [methyl-^3^H] uridine (Moravek Biochemicals; MT-6036) was added to each flask, and the cells were incubated for 1 hour at 37°C in a humidified atmosphere containing 5% CO_2_. Then the cells were collected on multiscreen GV filters under a vacuum and were subsequently washed twice with ice-cold 0.4N perchloric acid (70%) and 100% ethanol. The radioactivity in the acid-insoluble material retained on the filters was measured by scintillation counting, normalized to the cell count, and expressed as the percentage of DMSO-treated cells.

### 2.8 Measurement of nucleoside triphosphates

Nucleotides were extracted from 4 to 5 × 10^6^ cell pellets using perchloric acid, and the extracts were neutralized with potassium hydroxide. The neutralized extracts were applied to an anion-exchange Partisil 10 SAX column (4.6×250 mm, Whatman) and eluted at a flow rate of 1.5 mL/minute with a 50-minute concave gradient (curve 8; Waters Corp.) from 60% 0.005 M NH_4_H_2_PO_4_ (pH 2.8) and 40% 0.75M NH_4_H_2_PO_4_ (pH 3.8) to 100% 0.75 M NH_4_H_2_PO_4_ (pH 3.8). The column eluate was monitored with ultraviolet absorption at 262 nm, and the nucleoside triphosphates were quantitated by electronic integration with reference to external standards. The analogue triphosphate (8-Cl-ATP) was identified by comparing its retention profile and absorption spectrum with those of an authentic standard. The intracellular concentration of nucleotides contained in the extract was calculated from a given number of cells of a determined mean volume. The cell number was determined using a Coulter counter (Coulter Electronics). The equipment was attached to a channelizer, which was used to estimate the mean volume of cells in a particular cell population. This volume was used to quantitate the concentration of nucleotides. The calculation assumed that the nucleotides were uniformly distributed in a total cell volume. The lower limit of sensitivity of this assay was 10 pmol in an extract of 5 × 10^6^ cells, corresponding to a cellular concentration of 1 μM.

### 2.9 Seahorse XF Mito Stress Test assay

MEC-1 and HG-3 cells were seeded at a density of 1 × 10^6^ cells/mL and incubated for 24 hours with DMSO and CB-839 at equitoxic concentrations based on the IC_50_ values established during the MTS assay. For the combination treatments in the cell lines, venetoclax (5 μM), AZD-5991 (5 μM), or ibrutinib (1 μM), with or without addition of CB-839 (1 μM), were used. Samples from 5 patients with primary untreated CLL were collected as described above. The primary cells were treated with DMSO, venetoclax (10 nM), AZD-5991 (250 nM), or ibrutinib (1 μM), with or without CB-839 (1 μM). The primary CLL lymphocytes were incubated for 24 hours with CB-839 and ibrutinib; to avoid excessive cell death, venetoclax and AZD-5991 were not added until the last 6 hours of incubation. Samples were subjected to Seahorse analysis (Agilent) as previously described (28, 29). In brief, cells were washed twice with phosphate-buffered saline and resuspended in prewarmed (37 °C) Seahorse basal medium (Agilent) supplemented with 1 mM pyruvate, 2 mM glutamine, and 5 mM glucose (pH 7.4). Next, cells (175 μL) were suspended at a density of 0.5- to 1 × 10^6^ cells/mL for the cell lines and 5 × 10^6^/mL for the primary CLL primary lymphocytes. Then the cells were plated in at least 3 to 4 replicates on 96-well Seahorse Cell Culture (Agilent) plates precoated with Cell-Tak (Corning) cell adhesive. Once plated, the cells were gently centrifuged at room temperature for 4 minutes at 1500 rpm, without a break. Experimental protocols were set up with Seahorse XF Pro Controller software (Agilent), which allows easy assay protocol creation. The OCR and extracellular acidification rate (ECAR) were determined using a Seahorse XF Pro analyzer (Agilent) according to the manufacturer’s instructions. The OCR and ECAR values were obtained at baseline and after injections of Seahorse XF Mito Stress Test Kit (Agilent) reagents: oligomycin (1.5 μM), FCCP (1.0 μM), and antimycin/rotenone (0.5 μM). All measurements were taken using Seahorse Analytics Wave Pro software (Agilent) and normalized to the cell numbers obtained at the assay initiation. Normalized data were then exported as GraphPad files and subjected to statistical analysis using GraphPad 9.0 software.

### 2.10 Autophagy assays

To evaluate autophagy, microtubule-associated protein 1A/1B–light chain 3 (LC3) I to LC3II conversion and p62 protein degradation were measured using an immunoblot analysis as described below. Additionally, fluorescently labeled LC3 puncta were visualized using fluorescent imaging analysis. After fixation, cells were stained with anti-LC3A/B antibody (dilution 1:400) and secondary rabbit antibody (dilution 1:200) and analyzed using fluorescence confocal microscopy. Puncta were counted per 20 cells per condition, and an unpaired, 2-tailed Student *t*-test was performed to compare DMSO versus CB-839 treated cells. The antibodies used are listed in **Supplemental Table 2**. The autophagy-inhibitor bafilomycin A1 (100 nM) and the autophagy-inducer rapamycin (10 μM) were used as negative and positive controls, respectively.

### 2.11 Western blotting

CLL cells were lysed in a radio-immunoprecipitation assay buffer (Millipore-Sigma) with cOmplete mini protease inhibitor tablets (Roche) and were processed to extract the total protein. The protein concentration was quantified using a Pierce BCA Protein Assay Kit (Thermo Fisher Scientific) per the manufacturer’s instruction. Immunoblots were performed with 20 to 30 µg of cellular protein extracts, run on criterion protein gels (4-12% gradient gels; Bio-Rad) using a 3-(N-morpholino) propane-sulfonic acid running buffer (Bio-Rad), and transferred onto polyvinylidene difluoride membranes (Bio-Rad). Blots were cut into slices, incubated with specific antibodies, and visualized with the Odyssey Infrared Imaging System (LI-COR Biosciences). The antibodies used are listed in **Supplemental Table 2**. Secondary antibodies were chosen according to the species of origin of the primary antibodies.

The signal intensity of each band of target protein was measured and normalized to an internal loading control (either b-actin or vinculin) using Image Studio software (LI-COR Biosciences).

### 2.12 Statistical analysis

Data are expressed as the mean ± the standard error of the mean. Comparisons between 2 values were made using an unpaired, 2-tailed Student *t*-test unless otherwise specified. One-way ANOVA test (1-way analysis of variance test) was used to compare three or more independent treatment groups. A *P* value less than.05 was considered statistically significant. All graphs were generated using GraphPad Prism software. Drug synergism was measured using the Loewe model with Combenefit software (Cancer Research UK Cambridge Institute), and the combination index was calculated using CompuSyn software (ComboSyn) (30, 31).

## 3 Results

### 3.1 CB-839 treatment inhibits glutathione synthesis and the conversion of glutamine to glutamate in CLL cell lines

**Figure 1:**
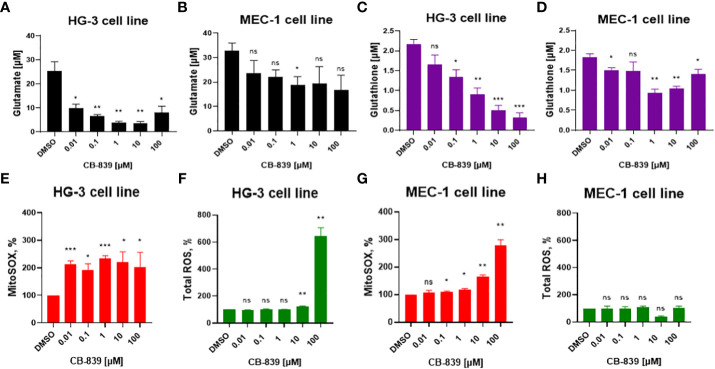
**(A, B)** The inhibition of glutaminase-1 (GLS-1), **(C, D)** the decline in glutathione levels, and changes in **(E, G)** mitochondrial superoxide (MitoSOX) and **(F, H)** total cellular reactive oxygen species (ROS) were evaluated in HG-3 and MEC-1 cells. Cells were treated with CB-839 (0.01-100 μM) for 72 hours, and the levels of these metabolites were determined using assay kits as described in the Methods section. ROS levels were evaluated per the manufacturer’s protocol after 4 hours of treatment; superoxide (SOX) levels measured after 24 hours of incubation. Data represent the mean ± the standard error of the mean for 3 biologically separate experiments. DMSO, dimethyl sulfoxide; ns, not significant. *P < 0.05; **P < 0.01; ***P < 0.001.

Initially, we investigated CB-839’s functional inhibition of GLS-1 (**Figures 1A, B**). The enzymatic activity of GLS-1 was evaluated by measuring the intracellular glutamate concentration in the presence of 0.01 μM to 100 μM of CB-839. At these concentrations, CB-839 decreased the intracellular glutamate levels by 48% in the MEC-1 cell line and by 86% in the HG-3 cell line compared to the controls. CB-839 also significantly reduced the conversion of glutamine to glutamate at 10 nM for the HG-3 cell line and at 1 μM for the MEC-1 cell line after 72 hours of incubation.

Since glutamate is one of the main precursors of glutathione, the impact of glutamate depletion on glutathione synthesis was assessed (**Figures 1C, D**). Inhibition of GLS-1 starting at 1 μM of CB-839 was enough to reduce the mean glutathione concentration by at least 51% in both the HG-3 and MEC-1 cell lines compared to the controls.

### 3.2 CB-839 exposure leads to mitochondrial ROS accumulation in CLL cells

Considering that glutathione is the main antioxidant that provides protection from excessive ROS production, we evaluated the consequences of glutathione synthesis inhibition on cellular ROS for 4 hours and mitochondrial SOX accumulation for 24 hours after incubation with CB-839 (**Figures 1E, G**). CB-839 treatment increased mitochondrial SOX in both cell lines at relatively low concentrations, starting at 10 nM for the HG-3 cell line and 100 nM for the MEC-1 cell line. However, the cellular ROS levels remained stable, and at higher concentrations of CB-839, only the HG-3 cell line demonstrated an increase in cellular ROS (**Figures 1F, H**). Overall, the HG-3 cell line was more sensitive to the inhibition of glutathione synthesis and required lower CB-839 concentrations to induce ROS overproduction.

To determine if ROS accumulation is a universal consequence of glutathione depletion in CLL cells, we treated the MEC-1 and HG-3 cell lines with BSO, a γ-glutamyl cysteine synthetase inhibitor. The CLL cells were sensitive to BSO treatment, and a dose-dependent decrease in the glutathione concentration was observed (**Supplemental Figure 2A**). However, no significant ROS accumulation (neither mitochondrial SOX nor cellular ROS) was detected upon BSO treatment (**Supplemental Figure 2B**). These findings suggest that CB-839 may induce alternative mechanisms of mitochondrial SOX accumulation.

### 3.3 Glutaminase inhibition affects mitochondrial respiration and energy metabolism in CLL cell lines but can be successfully compensated for by glycolysis

**Figure 2:**
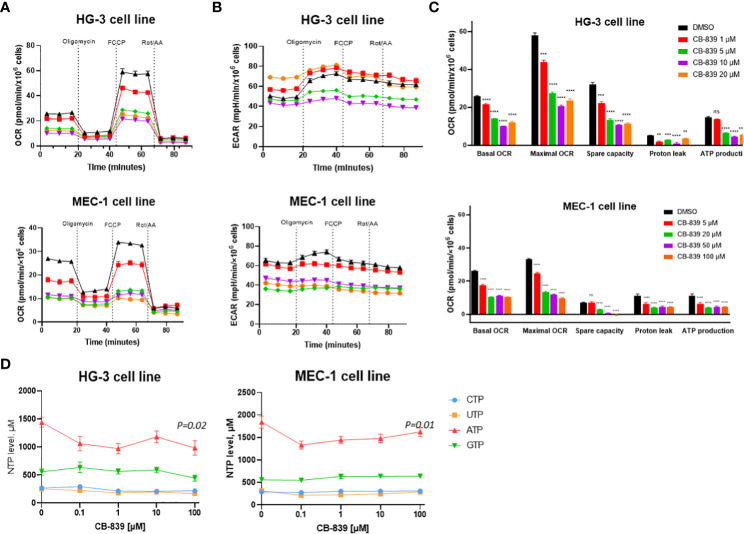
CB-839 affected mitochondrial respiration and bioenergetics in the HG-3 and MEC-1 cell lines. Representative **(A)** oxygen consumption rate (OCR) and **(B)** extracellular acidification rate (ECAR ECAR) curves of untreated cells versus cells treated with CB-839 in equitoxic concentrations in the HG-3 (top) and MEC-1 (bottom) cell lines. **(C)** Changes in the basal OCR, maximal OCR, spare capacity, proton leakage, and ATP-linked production under CB-839 treatment in the HG-3 (top) and MEC-1 (bottom) cell lines. **(D)** CB-839–induced depletion of the ATP pool in the HG-3 and MEC-1 cell lines was detected using high-performance liquid chromatography. The significance of the results was evaluated using a 1-way analysis of variance test. ATP, adenosine triphosphate; CTP, cytidine triphosphate; DMSO, dimethyl sulfoxide; GTP, guanosine triphosphate; ns, not significant; Rot/AA, rotenone/antimycin A; UTP, uridine triphosphate. **P < 0.01; ***P < 0.001; ****P < 0.0001.

In the HG-3 and MEC-1 cell lines, CB-839 treatment caused dose-dependent decreases in the OCR, including the basal OCR, maximal respiration rate, and spare respiratory capacity. This finding indicated a profound impairment of ATP production, reflected by decreases in the OCR-linked ATP levels (**Figures 2A, C**). Mechanistically, these data, together with observed decrease in proton leakage, indicated profound changes in mitochondrial function leading to energy depletion. However, we found that the CLL cells that were initially metabolically quiescent, as indicated by their low OCR and ECAR) had a strongly upregulated ECAR when they were subjected to oligomycin treatment. This finding indicated the cells’ propensity to use glycolysis when under stress. Notably, that MEC-1 cells had higher basal level of ECAR compared to HG-3 cell line, which may contribute to their lower sensitivity to OXPHOS inhibition (**Figure 2B**). Consistent with its disruptions of mitochondrial respiration reflected in the cells’ OCR measurements, CB-839, starting at low concentrations of 0.1 to 1 μM, also decreased the ATP pool in the HG-3 (P = 0.02) and MEC-1 (P = 0.01) cell lines, as measured using high-performance liquid chromatography (**Figure 2D**).The levels of other nucleotides remained stable at all concentration points.

### 3.4 CB-839 promotes a dose-dependent decrease in CLL cell lines’ proliferation but does not promote apoptosis

**Figure 3:**
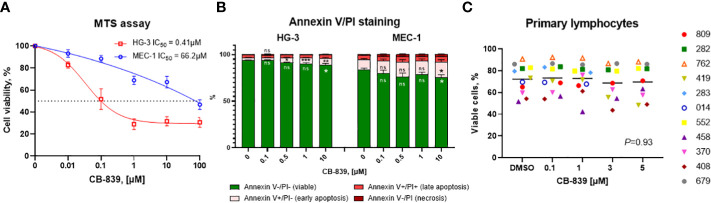
The characterization of the *in vitro* growth inhibitory effects of CB-839 (concentration range, 0.01-100 μM) after 72 hours. The percentages of viable cells from the HG-3 and MEC-1 cell lines as determined using **(A)** an MTS colorimetric assay and **(B)** annexin V/propidium iodide (PI) staining. **(C)** The percentages of viable primary lymphocytes in CB-839–treated samples after 72 hours as determined using flow cytometry (n = 11; *P* = 0.93). The data are presented as the mean ± the standard error of the mean. Values were calculated from 3 independent experiments, and each experiment was performed 3 times. The significance of the results was evaluated using a 1-way analysis of variance test. Open symbols represent patients without the immunoglobulin heavy chain (*IGHV*) mutation, and solid symbols represent those with the mutation. The numbers next to the open and solid symbols are patient numbers.

Next, we investigated the impact of CB-839 treatment on biological effects such as cell proliferation and cell death. In the HG-3 and MEC-1 cell lines, the inhibition of GLS-1 induced a dose-dependent decrease in cell proliferation, as detected using an MTS assay. The IC_50_ that caused a 50% decrease in cell viability was notably higher (0.41 μM for HG-3 cells and 66.2 μM for MEC-1 cells) than the concentration of CB-839 required for functional GLS-1 inhibition (**Figure 3A**). Thus, the decrease in cell viability may be explained by the off-target effects of CB-839 at high concentrations. Furthermore, flow cytometry measurements revealed no significant increases in the percentages of early-apoptotic or late-apoptotic cells upon CB-839 treatment. For the dose of CB-839 10 μM, on average, 89% (range 79.2-92.7%) of HG-3 cells and 75% (range, 63-85,8%) of MEC-1 cells remained viable after 72 hours of incubation (**Figure 3B**). While BCL-2 protein expression upon CB-839 treatment remained stable even at maximal concentrations of 100 μM, MCL-1 protein levels increased in both cell lines in cells incubated with CB-839 compared with control cells, indicating that the HG-3 and MEC-1 cells had an MCL-1 protein dependency in response to apoptotic stimuli (**Supplemental Figure 3**). Altogether, we observed that CB-839 had cytostatic rather than cytotoxic effects on CLL cells.

To determine if the inhibition of proliferation is mediated by impaired DNA or RNA synthesis, we assessed the inhibition of DNA and RNA synthesis by the incorporation of exogenous radioactive thymidine and uridine (**Supplemental Figure 4**). In the HG-3 and MEC-1 cell lines, after the normalizing radioactivity (in disintegrations per minute) to the cell counts, we did not observe any decreases in thymidine or uridine incorporation after 72 hours of incubation with CB-839 at any dose.

### 3.5 CB-839 induces autophagy in CLL cell lines

**Figure 4:**
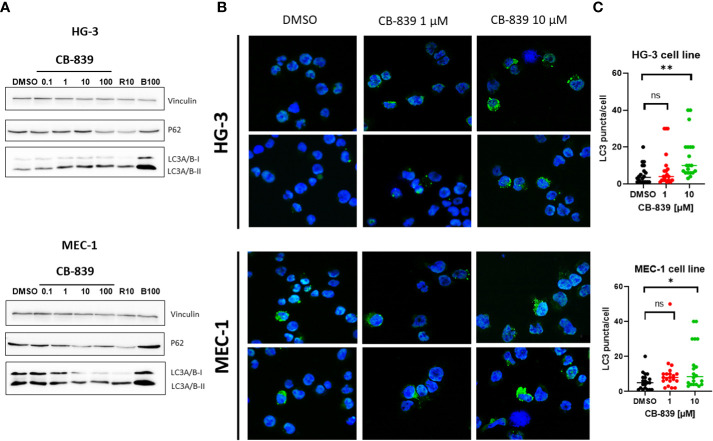
CB-839 induced autophagy in the HG-3 and MEC-1 cell lines. **(A)** An immunoblot assay revealed increased conversion of light chain 3 (LC3)-I to LC3-II and a decrease in p62 expression in the treated cells compared with the untreated cells. **(B)** CB-839 increased the number of green puncta in the treated cells compared with the control (dimethyl sulfoxide [DMSO]-treated) cells (12.6X). **(C)** Quantitation of LC3 puncta in HG-3 and MEC-1 cell lines after treatment with CB-839. Number of puncta in figure 4B were quantitated. 20 cells in each condition were used to count puncta and data were expressed per cell. Rapamycin (R10; 10 μM) and bafilomycin A1 (B100; 100nM) were used as positive and negative controls. ns, not significant. *P < 0.05; **P < 0.01.

Because glutamine flux regulates mammalian target of rapamycin complex 1’s kinase activity, which is responsible for autophagy, we assessed the effects of glutaminase inhibition on autophagy as an alternative mechanism of CB-839–mediated cell death (32–35). We found that treating HG-3 and MEC-1 cells with CB-839 increased LC3-I to LC3-II protein conversion and p62 protein degradation (**Figure 4A**). Notably, the HG-3 cells had a relatively high basal level of autophagy. These results were validated using fluorescent miscroscopy and immunocytochemical staining (**Figure 4B**), which showed that CB-839 induced the accumulation of LC3 puncta in HG-3 and MEC-1 cells. More LC3 puncta were observed in the cytoplasm of cells treated with CB-839 (10 μM) than in that of untreated control cells (P = 0.002 for HG-3 cells; P = 0.01 for MEC-1 cells; **Figure 4C**). Because autophagy may serve as a protective mechanism for CLL cells (36), we then tested CB-839 in combination with the autophagy inhibitor chloroquine at a concentration of 50 μM (**Supplemental Figure 5**). Despite chloroquine’s successful inhibition of LC3 conversion, we observed no significant induction of apoptosis after addition of CB-839 upon evaluation of the cells using annexin V/PI flow cytometry staining. Collectively, these data suggested the limited activity of CB-839 monotherapy in CLL cell lines.

### 3.6 CB-839 alone has limited effects on primary CLL cells

To evaluate the effect of CB-839 monotherapy on primary CLL cells, lymphocytes were isolated from 11 patient samples and incubated with 100 nM, 1 μM, 3 μM, and 5 μM of CB-839 for 24 hours. Nine patients had a mutated immunoglobulin heavy chain (IGHV) gene, which is associated with a favorable prognosis, and 2 had an unmutated gene. Since primary CLL cells have a very limited growing capacity, an apoptosis assay was performed after 24 hours of incubation. In line with previous observations in CLL cell lines, CB-839 did not cause significant apoptosis in the cells, and the level of double-negative events as determined using annexin V/PI staining remained stable in all the treatment groups (P = 0.93). Even in cells from the patients with the prognostically favorable IGHV mutation, CB-839 did not induce cell death (**Figure 3C**). In addition, glutaminase inhibition had no effect on the bioenergetics of the primary CLL cells. Specifically, the cells’ basal OCR, maximal OCR, and spare respiratory capacity were not impacted after 24 hours of incubation with 1 μM CB-839 (**Supplemental Figure 6**). Similar to the CLL cell lines, 6 patient samples were assessed for nucleoside triphosphate levels after 8 hours of treatment. Unlike the cell lines, the primary cells showed no depletion of the nucleoside triphosphate pool; the ATP, cytidine triphosphate, uridine triphosphate, and guanosine triphosphate levels were constant for all concentrations of CB-839 because of the slow proliferation rate of the primary cells *in vitro* (**Supplemental Figure 7**).

### 3.7 CB-839 affects oxygen consumption and cell proliferation, and induces apoptosis in combination with venetoclax, AZD-5991, but not ibrutinib in cell lines

**Figure 5:**
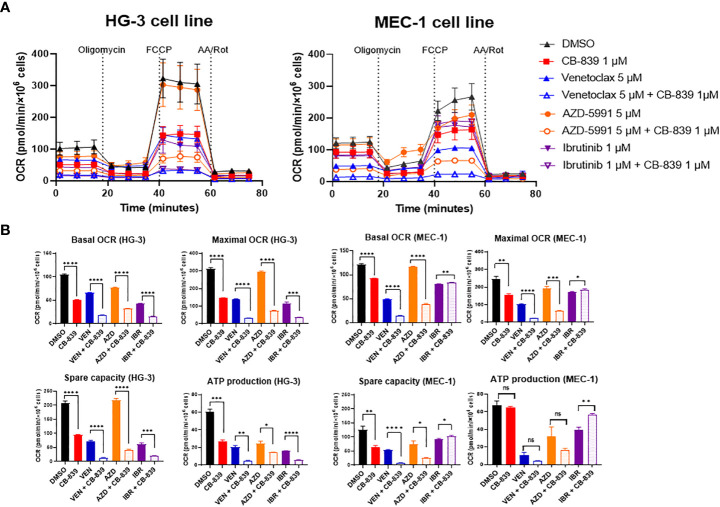
The inhibition of mitochondrial respiration in the HG-3 and MEC-1 cell lines upon treatment with CB-839 in combination with venetoclax, AZD-5991, and ibrutinib. **(A)** Oxygen consumption rate (OCR) curves for the control (dimethyl sulfoxide [DMSO]-treated), alone, and combination treatment groups. **(B)** Changes in basal OCR, maximal OCR, spare capacity, and ATP-linked production under CB-839 treatment in the HG-3 (left) and MEC-1 (right) cell lines. AA/Rot, rotenone/antimycin A; AZD, AZD-5991; IBR, ibrutinib; ns, not significant; VEN, venetoclax. *P < 0.05; **P < 0.01; ***P < 0.001; ****P < 0.0001.

To improve the bioenergetic effects of glutaminase inhibition on the HG-3 and MEC-1 cell lines, CB-839 was tested in combination with CLL-specific drugs. (**Figures 5A, B**) Compared to cells treated with CB-839, venetoclax and AZD-5991 treated cells exhibited greater suppression of mitochondrial respiration. In the MEC-1 cell line, using ibrutinib alone was not as successful at blocking cell respiration as using CB-839. Combining ibrutinib and CB-839 did not lead to a greater decrease in OCR. Notably, that OCR-linked ATP production was barely impacted in the MEC-1 cell line. Nevertheless, when combining CB-839 with venetoclax and AZD-5991, there was a significant drop in other OCR parameters such as basal OCR, maximal OCR, and spare respiratory capacity when compared to monotherapy. For the HG-3 cell line, using CB-839 and ibrutinib together had a stronger effect on inhibiting OCR than using either drug alone.

**Figure 6:**
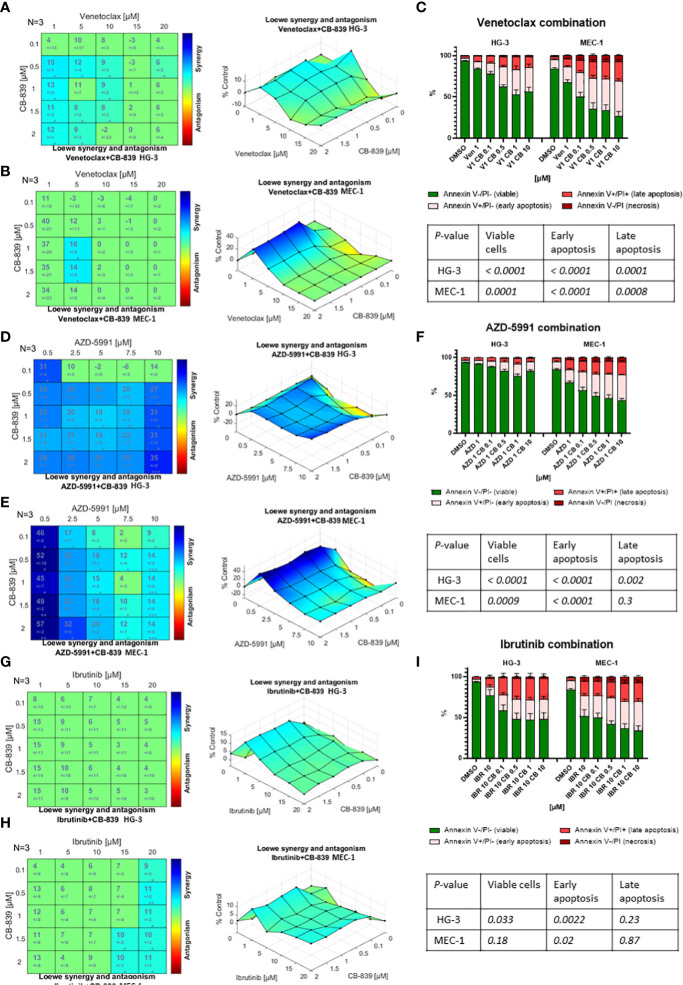
The drug interactions in the HG-3 and MEC-1 cell lines were visualized using Combenefit software. The colored matrices show the synergy scores for the drug combinations, and the surface plots show data representative of 3 independent experiments. When given in combination therapy, CB-839 showed **(A, B)** a narrow synergistic concentration window with venetoclax, **(D, E)** synergism with AZD-5991, and **(G, H)** no synergism with ibrutinib. **(C, F, I)** The Loewe model data were confirmed by an evaluation of apoptosis using annexin/propidium iodine (PI) staining and flow cytometry. Flow cytometry data were analyzed using 1-way analysis of variance testing. Cells were treated with dimethyl sulfoxide (DMSO; controls), monotherapy (venetoclax [VEN], AZD-5991 [AZD], and ibrutinib [IBR]), or combination therapy with CB-839 (CB). *P* values are given in the tables below each graph. The data are presented as the mean ± the standard error of the mean for 3 independent experiments, and each experiment was performed 3 times.

Because CB-839 alone had a limited effect on cell proliferation and apoptosis, we examined whether the metabolic changes associated with this drug would sensitize the CLL cell lines and primary cells to the targeted therapeutics. The sensitization potential of CB-839 on cells treated with venetoclax (**Figures 6A–C**), AZD-5991 (**Figures 6D–F**), or ibrutinib (**Figures 6G–I**) was assessed using the Loewe additivity model and validated using annexin V/PI staining. Despite its higher sensitivity towards CB-839, the HG-3 cell line was less responsive to all drug combinations than the MEC-1 cell line (**Supplemental Table 3**). In the MEC-1 cell line, the synergistic effect of CB-839 and venetoclax was noted only for a narrow concentration window (1-1.5 μM of CB-839 combined with 5 μM of venetoclax). In the HG-3 cell line, the CB-839 and venetoclax combination did not demonstrate strong synergism. Generally, the HG-3 and MEC-1 cells were resistant to AZD-5991 treatment. A high concentration of single-agent AZD-5991 was needed to achieve the IC_50_. However, the addition of CB-839 sensitized cells to MEC-1 inhibition and showed synergism; the combination index was less than 0.2 for all effective doses, with 19.8-fold and 5-fold shifts in the IC_50_ (0.5 μM for HG-3 and 0.38 μM for MEC-1 cell line) (**Supplemental Table 3**, **Supplemental Figure 8**). Finally, the combination of CB-839 and ibrutinib produced generally additive rather than synergistic effects in both cell lines.

### 3.8 CB-839 did not improve cytotoxicity in combination with venetoclax, AZD-5991, and ibrutinib in primary CLL lymphocytes

**Figure 7:**
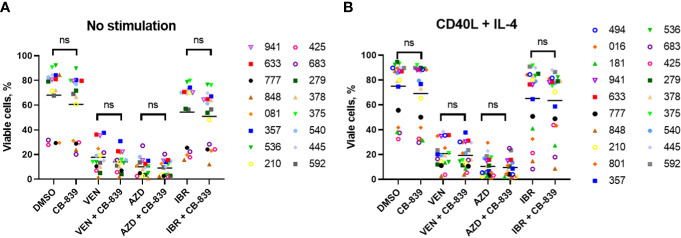
**A** total of 19 patient-derived chronic lymphocytic leukemia samples were incubated for 72 hours **(A)** without (n = 16) and **(B)** with (n = 19) the proliferation-inducing cytokines CD40 ligand (CD40L) and interleukin-4 (IL-4). Both groups were treated with venetoclax (VEN; 10 nM), AZD-5991 (AZD; 250 nM), and ibrutinib (IBR; 1 μM), with or without addition of CB-839 (500 nM), for 72 hours. Apoptosis was quantified using flow cytometry and annexin/propidium iodide (PI) staining. For each comparison of a drug combination or control (dimethyl sulfoxide [DMSO]) versus CB-839 alone, an unpaired, 2-tailed Student *t*-test was performed. The numbers next to the open and solid symbols are patient numbers. ns, not significant.

We then tested these drug combinations in the primary CLL cells, both in the native condition (**Figure 7A**) and in media supplemented with cytokines (CD40 ligand and interleukin-4) to mimic the microenvironment and maintain viability (**Figure 7B**). Since venetoclax and AZD-5991 rapidly induce apoptosis in CLL cells, these compounds were added in the last 24 hours of incubation to minimize single-drug cytotoxicity. Nineteen patient samples were incubated for 72 hours with CB-839 (0.5 μM) and with venetoclax (10 nM), AZD-5991 (250 nM), or ibrutinib (1 μM) with or without CB-839. For venetoclax alone, a median of 82.2% of cells died, and the percentage was similar (85.9%) after the addition of CB-839 (P = 0.28). CLL cells were sensitive to AZD-5991 alone and in combination with CB-839 (90% of cell death in both groups; P = 0.67). Ibrutinib treatment resulted in a lower level of cell death (45.6%), which was not modulated by the addition of CB-839 (49.1%; P = 0.67; **Figure 7A**). The stimulation of the primary cells with CD40 ligand and interleukin-4 caused no changes in the cells’ response to drug treatment. Likewise, the addition of CB-839 to venetoclax, AZD-5991, or ibrutinib along with cytokine stimulation did not increase the apoptosis rate (P = 0.67, P = 0.71, and P = 0.85, respectively; **Figure 7B**). The combined effect of these drugs was similar in both the IGHV-mutated and unmutated CLL subsets.

**Figure 8:**
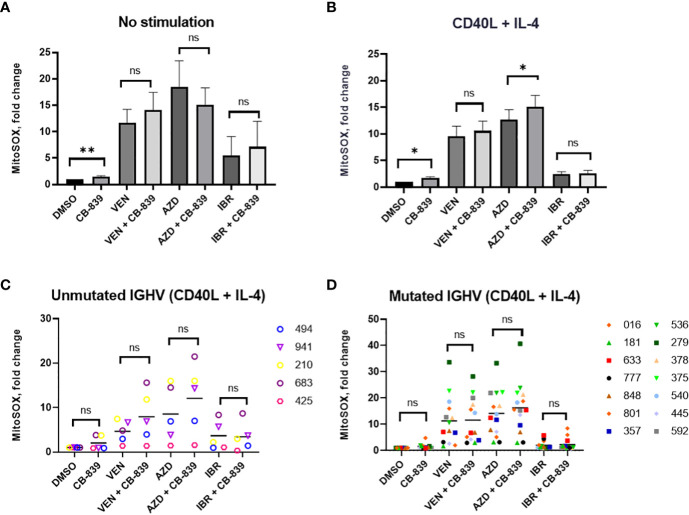
**(A, B)** Both CD40 ligand (CD40L) and interleukin-4 (IL-4)–stimulated and unstimulated primary chronic lymphocytic leukemia lymphocytes increased their mitochondrial reactive oxygen species production in response to 72 hours of CB-839 treatment as revealed by mitochondrial superoxide red fluorescence. **(C, D)** The immunoglobulin heavy chain (*IGHV*) mutational status had no impact on venetoclax (VEN), AZD-5991 (AZD), and ibrutinib (IBR) sensitization by CB-839. Data represent the mean ± the standard error of the mean. For each comparison of a drug combination or control (dimethyl sulfoxide [DMSO]) versus CB-839 alone, an unpaired, 2-tailed Student *t*-test was performed. Open symbols represent patients without the *IGHV* mutation, and solid symbols represent those with the mutation. The numbers next to the open and solid symbols are patient numbers. ns, not significant. *P < 0.05; **P < 0.01.

The treatment of primary CLL lymphocytes with CB-839 alone increased mitochondrial superoxide production in both unstimulated (n = 15) and stimulated (n = 19) conditions (P = 0.006 and P = 0.02, respectively; **Figures 8A, B**). Treatment with the targeted agents alone also increased mitochondrial superoxide production; we observed a 11.7-fold change for venetoclax, a 18.4-fold change for AZD-5991, and an 5.5-fold change for ibrutinib. Notably, cells treated with CD40 ligand and interleukin-4 stimulation had lower ROS levels than unstimulated cells. *However, this ROS decrease did not impact the drug-related apoptosis rate. Cytokine stimulation sensitized primary lymphocytes to AZD-5991 and CB-839 combination, compared to AZD-5991 alone (*P*=0.02)* Cells treated with venetoclax and ibrutinib combination with CB-839 did not have significantly increased mitochondrial superoxide accumulation compared to single drug. Cells’ IGHV mutational status did not have any impact on ROS production (**Figures 8C, D**). One patient with the combination of del(17p) and chromosome 12 trisomy had an abnormally high level of mitochondrial superoxide after AZD-5991 and ibrutinib treatment (a 551-fold increase while on ibrutinib alone and a 74-fold increase while on combination treatment with ibrutinib and CB-839; **Supplemental Figure 9**). Unlike the HG-3 and MEC-1 cell lines, the patients’ primary lymphocytes did not respond metabolically to CB-839 given in combination with venetoclax, AZD-5991, or ibrutinib. No significant decrease in the OCR was observed in 5 Seahorse-analyzed samples (**Supplemental Figure 6**).

**Figure 9:**
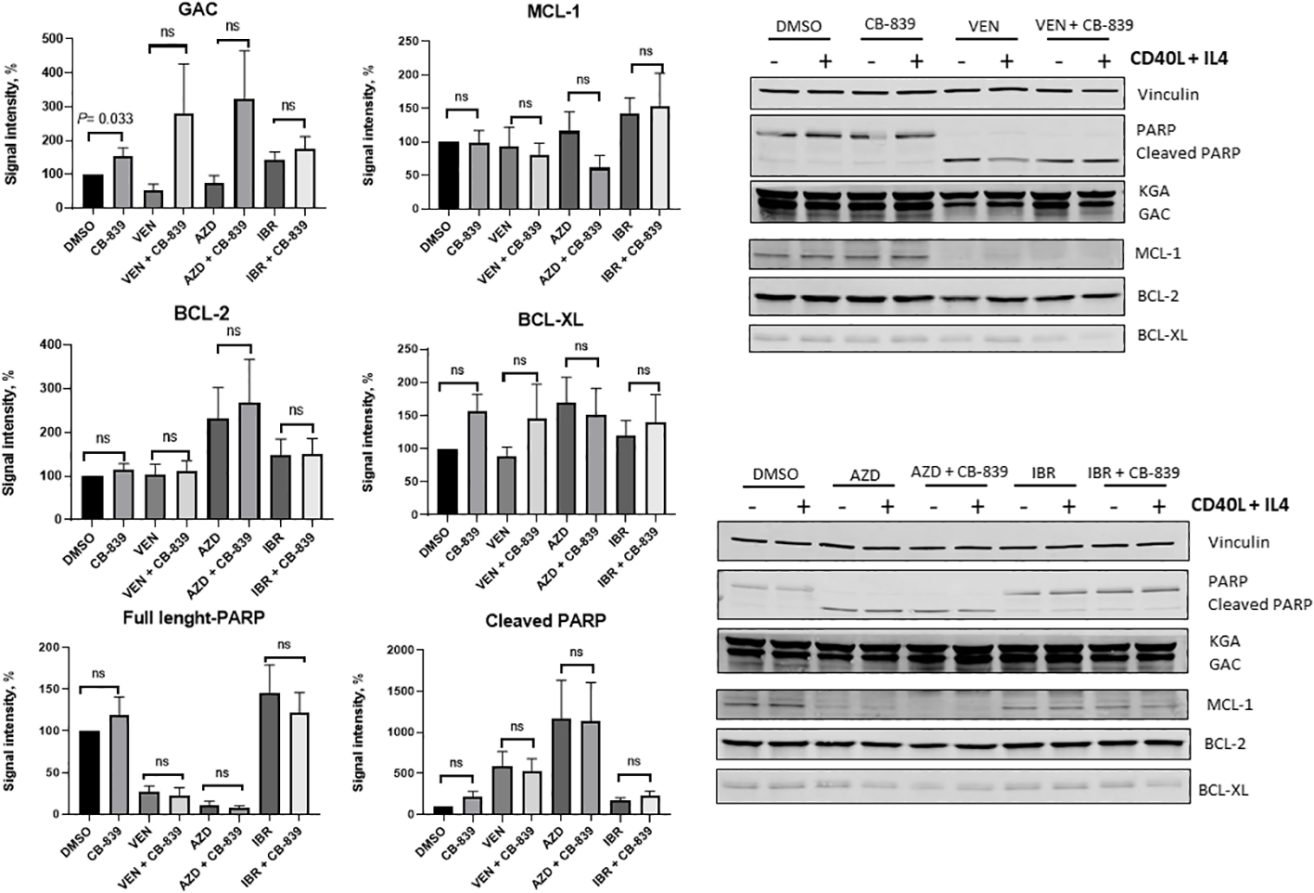
Summary plots of Western blotting analysis and quantifications of glutaminase C (GAC), myeloid cell leukemia-1 (MCL-1), B-cell lymphoma-2 (BCL-2), B-cell lymphoma–extra-large (BCL-XL), and PARP (full-length and cleaved) protein expression in 10 patient samples after CB-839 treatment alone and in combination with venetoclax (VEN), AZD-5991 (AZD), and ibrutinib (IBR). Bar plots represent groups supplemented with CD 40 ligand (CD40L) and interleukin-4 (IL-4). Data are shown as the mean ± the standard error of the mean. Signal intensity was normalized to the loading control (vinculin, β -actin). For each comparison of a drug combination or control (dimethyl sulfoxide [DMSO]) versus CB-839 alone, an unpaired, 2-tailed Student *t*-test was performed. KGA, kidney glutaminase; ns, not significant.

Eleven samples were available for the immunoblot assay (**Figure 9**). The expression levels of PARP (full length and cleaved), MCL-1, BCL-2, and BCL-XL were not affected by the addition of CB-839. Glutaminase C expression was increased in the CB-839-treated group (P = 0.033). There was a trend of increasing glutaminase C expression in all combinations treated with CB-839, but the trend was not statistically significant. CD40 ligand and interleukin-4 stimulation increased the overall expression of proteins, but the differences between single-agent venetoclax, AZD-5991, and ibrutinib and their combinations with CB-839 remained nonsignificant.

## 4 Discussion

Glutamine, an amino acid needed for protein synthesis and for cellular proliferation, is converted to glutamate by glutaminase, and the disruption of cancer metabolism through glutamine inhibition has been an attractive target for solid and hematological tumor therapies for many years. The consequences of glutaminase inhibition have been studied in solid cancers with promising results (20–22), but for hematological malignancies, data are still limited.

Although data on the use of glutaminase inhibitor CB-839 in CLL are not available, Parlati et al. (26) investigated CB-839’s effects on 18 hematological malignancy cell lines, including multiple myeloma, acute leukemia, and diffuse large B-cell lymphoma cell lines. Sixteen of these cell lines were sensitive to CB-839 treatment and had an antiproliferative IC_50_ of less than 100 nM (range, 2-72 nM). Most other CB-839 *in vitro* studies have focused on CB-839’s pharmacological interactions and uses in combination therapy rather than its use as a single agent. The fixed concentrations of CB-839 used in these studies (up to 1 μM for AML (10, 11, 37) and 5 μM for multiple myeloma (24) were used without evaluation of the IC_50_. In our experiments, the concentration of CB-839 needed for effective, functional glutaminase inhibition varied from 10 nM to 1 μM, and the antiproliferative IC_50_ was determined as 0.41 μM for the HG-3 cell line and 66.2 μM for the MEC-1 cell line.

The *in vitro* biological effects of CB-839 have been tested more extensively in multiple myeloma (24), AML (10, 11), and T-cell acute lymphoblastic leukemia cell lines and primary blasts (29). In multiple myeloma, CB-839 as a single agent was not efficient, but the main purpose of the research was the discovery of the mechanisms that help to overcome resistance to carfilzomib. CB-839 inhibited mitochondrial respiration in multiple myeloma cells and restored sensitivity to proteasome inhibitor (24). In AML cell lines and primary blasts, CB-839 decreased cell viability, blocked glutamine use, and sensitized leukemic cells to priming with venetoclax (10, 11). Similar effects were observed in models of glutamine-dependent T-cell acute lymphoblastic leukemia, in which CB-839 alone at a concentration of 1 μM was strongly viable, reduced the OCR, and produced synergistic effects when cells were treated in combination with inhibitors of mitochondrial complex I (29).

CLL is a heterogeneous disease, and the different subtypes of CLL have diverse metabolic requirements. Previous studies have shown that more aggressive variants demonstrate an enhanced reliance on OXPHOS. In CLL, a higher OCR is associated with prognostically unfavorable factors such as advanced stage, unmutated *IGHV* status, ZAP-70 positivity, and high level of β2-microglobulin as well as development of drug resistance (17) and an increased risk of Richter’s transformation (38). However, no such differences have been seen in cytogenetic variant samples (1).

Drugs that impair energy metabolism have already been tested in CLL. The OXPHOS inhibitor IACS-010759 depleted the intracellular nucleoside triphosphate pool and caused minor cell death; however, compensatory glycolysis and glucose uptake were observed in response to OXPHOS inhibition (3). The attempt to induce energy starvation by reducing the ATP pool with halogenated adenosine (8-Cl-Ado) resulted in global RNA synthesis inhibition and a decrease in MCL-1 protein expression (6). MCL-1 enhances survival and protects CLL cells from mitochondria-mediated death (39). This explains the increased cell death in transcriptionally challenged CLL cells (6). Also, the activation of fatty acid oxidation was discovered as an alternative way to feed the TCA cycle upon ibrutinib treatment, which decreases glutamine availability (19). Thus, it appears that CLL cells show plasticity in response to various metabolomic interventions.

Investigators have paid special attention to del(11q)-positive CLL because the *ATM* gene found in that region plays a meaningful role in metabolism and ROS regulation (40). Although cytogenetic abnormalities did not impact OCR values (1), comparison of del(11q)-positive CLL with wild-type CLL showed that the 2 varieties of CLL had different metabolic backgrounds. The del (11q) CLL cells were more sensitive to glutaminase inhibition than the wild-type CLL cells. In addition, compound 968 (a GLS-1 inhibitor) was cytotoxic for the del(11q) CLL cells, in which ROS levels were increased upon GLS-1 inhibition. Glutamine and glutamate uptake was not affected by compound 968, suggesting that CLL lymphocytes are able to maintain constant concentrations of these metabolites (4). Nevertheless, compound 968 has different pharmacological characteristics than CB-839 and is less effective in disrupting glutaminyls (41).

We investigated the inhibition of glutaminase in CLL cell lines and treatment-naïve primary CLL lymphocytes and the consequences for cell growth and survival. CB-839 efficiently inhibited GLS-1’s function in the CLL cell lines, but higher doses were required to affect cell proliferation. Metabolically, CB-839 effectively inhibited OCR and ATP production in both cell lines, providing evidence of CB-839’s mechanism of action in CLL cells. However, the CLL cells demonstrated a compensatory increase in glycolysis in response to oligomycin-induced stress. The main effect of CB-839 was cytostatic, and no significant induction of apoptosis was observed. Moreover, using a thymidine and uridine incorporation assay, we observed a compensatory increase in DNA and RNA synthesis. Primary cells from CLL patients were unresponsive to CB-839–mediated apoptosis induction and mitochondrial respiration inhibition.

As a mechanism of low cell death, the autophagy process was investigated in HG-3 and MEC-1 cells. We detected increased basal autophagy in the HG-3 cell line, but after incubation with 10 μM of CB-839, both cell lines demonstrated increased LC3 conversion and p62 degradation. These findings suggested that autophagy may support the cellular stress response in CLL cells. Accordingly, we tested CB-839 in combination with the autophagy inhibitor chloroquine and examined the apoptosis rate. Unfortunately, the inhibition of autophagy was not enough to eliminate cells’ resistance to CB-839. Generally, MEC-1 cells were less sensitive to CB-839 than were wild-type HG-3 cells, potentially because of the MEC-1 cells’ complex karyotype, including del(17p). It has been demonstrated that the *TP53* gene, located on chromosome 17, contributes to the maintenance of mitochondrial integrity, and that *TP53* deficiency can be compensated for by an increase in aerobic glycolysis and an enhancement of mitochondrial mass (42, 43). Only 4 patients in our sample carried the del(17p) mutation; thus, there were too few patients for an evaluation of the sensitivity of del(17p)-positive patients to glutaminase inhibition.

One of the biological effects of CB-839 is the inhibition of glutathione synthesis due to the depletion of glutamate as the pivotal precursor for glutathione synthesis. We observed a decrease in glutathione synthesis in both cell lines. The accumulation of predominantly mitochondrial SOX observed in CB-839–treated cells accompanied the impairment of mitochondrial glutaminase activity in the mitochondria and the depletion of the intermediates needed for glutathione synthesis. Even though we detected a decrease in glutathione after CB-839 therapy, glutathione depletion was not necessarily associated with ROS accumulation in CLL cells. BSO**’**s inhibition of glutathione synthesis did not result in ROS accumulation in the HG-3 and MEC-1 CLL cell lines. Glutathione helps eliminate SOX, but the reaction is relatively slow (44); thus, SOX may be removed *via* other pathways and antioxidants, such superoxide dismutase and glutathione peroxidase (45, 46). The effect of CB-839 on antioxidant defense systems require further studies. Moreover, the blockade of glutathione synthesis alone might not be sufficient to enhance oxidative stress and cell death, as the reduced glutathione pool might be partially regenerated from the oxidized glutathione pool (47). The ATP pool was slightly affected by CB-839 in the CLL cell lines. However, contrary to expectations, the primary lymphocytes maintained a stable level of ATP even after exposure to high CB-839 concentrations.

Because of the limited efficacy of CB-839 as a single agent, combinations of CB-839 with venetoclax, AZD-5991, and ibrutinib were assessed using the Loewe synergy model. Among all the combinations, the AZD-5991 and CB-839 combination showed solid synergism in antiproliferative activity and apoptosis induction. Venetoclax demonstrated only partial synergism with CB-839, and this synergism was more prominent in the MEC-1 cell line. Notably, CB-839 caused an increase of MCL-1 protein expression in the CLL cell lines, but the cells**’** BCL-2 expression remained stable. This phenomenon supports the observation that CLL cells exhibit increased sensitivity to CB-839 in combination with AZD-5991, the MCL-1 inhibitor; the combination eliminates MCL-1 protein**’**s protective effect. The addition of ibrutinib to CB-839 did not have any remarkable effect on cell proliferation and apoptosis, and no evidence of synergism was found for this combination.

For the primary cells, 2 subsets of CLL lymphocytes were tested: proliferating cells (supplemented with CD40 ligand and interleukin-4) and quiescent cells. The cytokine addition prolonged the CLL cells**’** survival and reduced the absolute ROS production. Notably, that CD40 ligand and IL-4 stimulation led to increase of mitochondrial sSOX in AZD-5991 combination with CB-839 compared to unstimulated cells. This finding supports previous observations of synergistic effect of GLS-1 and MCL-1 inhibition. However, cytokine supplementation did not change the pattern of CB-839–mediated effects when the drug was used alone rather than in combination with venetoclax and ibrutinib. Even though *IGHV* mutational status is the main biological prognostic factor for CLL, it did not impact the drug response.

Current investigations focused on the evaluation of CB-839 effects and synergism only during *in vitro. In vivo* studies are necessary to assess long-term exposure, preclinical safety, and the impact of the tumor microenvironment. Currently, clinical trials are investigating efficacy of CB-839 treatment in AML (NCT02071927) (48), myelodysplastic syndrome (NCT03047993) (49), non-Hodgkin lymphomas, and multiple myeloma (NCT02071888) (50) but the final results are yet to come. Therefore, preclinical data and data on the clinical relevance of CB-839 in CLL treatment are still unavailable.

## 5 Conclusions

Considering our results and those of previous studies, we suggest that CLL cells can efficiently rewire their amino-acid and glucose metabolism to evade glutaminase inhibition. On the basis of the present data, we suggest that CB-839 as a single agent may not be an option for patients with CLL. However, CB-839 in combination with other agents targeting multiple mechanisms may provide an additional strategy to overcome the metabolic adaptations in CLL. Further investigations evaluating these combinations *in vivo* are warranted.

## Data availability statement

The original contributions presented in the study are included in the article/**Supplementary Material**. Further inquiries can be directed to the corresponding author.

## Ethics statement

The studies involving human participants were reviewed and approved by MD Anderson Institutional Review Board. The patients/participants provided their written informed consent to participate in this study. Peripheral blood was obtained from patients who providedwritten informed consent as part of a protocol approved by theInstitutional Review Board of The University of Texas MDAnderson Cancer Center and in accordance with the Declarationof Helsinki.

## Author contributions

NT performed most of the experiments in the cell lines, coordinated the collection of patient samples, processed all samples, conducted experiments, analyzed data, and wrote most of the manuscript. MA performed the DNA/RNA synthesis experiments and nucleotide measurements. NB conducted the Seahorse experiments. JS-O’F performed the fluorescent microscopy experiments. GB conducted the Western blotting experiments (for autophagy). ZL provided her expert opinion on the autophagy evaluation and revised the experimental results. MK conceived the project and provided guidance and directions. VG conceived the project, analyzed data, and wrote portions of the manuscript. All authors commented on and critically revised the manuscript. All authors contributed to the article and approved the submitted version.
