# Supplementary material for: Preclinical investigations of the efficacy of the glutaminase inhibitor CB-839 alone and in combinations in chronic lymphocytic leukemia
Source: Front Oncol. 2023 May 9;13:1161254. doi: 10.3389/fonc.2023.1161254 (PMC10203524; doi:10.3389/fonc.2023.1161254)
Supplement: Supplementary file 1 [file DataSheet_1.docx]

Supplemental materials

**Supplemental Table 1. Patient characteristics.**

| **N** | **Patient ID #** | **White blood cell count,**  **× 10^9^/L** | **FISH result** | ***IGHV* status** | **ZAP-70 status** |
| --- | --- | --- | --- | --- | --- |
| **Group with 24 hours of incubation** | | | | | |
| 1 | 283 | 66.5 | del(13q) | Mutated | Neg |
| 2 | 809 | 46.2 | del(13q) | Mutated | Unk |
| 3 | 014 | 5.8 | del(11q) | Unmutated | Pos |
| 4 | 419 | 44.9 | del(13q) | Mutated | Neg |
| 5 | 762 | 23.5 | Tri12 | Unmutated | Pos |
| 6 | 679 | 40 | UNK | Mutated | Pos |
| 7 | 552 | 70.8 | del(13q) | Mutated | Unk |
| 8 | 458 | 109.7 | del(13q) | Mutated | Neg |
| 9 | 370 | 23.5 | del(13q) | Mutated | Neg |
| 10 | 408 | 48.8 | Tri12, del(13q) | Mutated | Pos |
| 11 | 828 | 38.6 | neg | Mutated | Neg |
| **Group with 72 hours of incubation** | | | | | |
| 1 | 016 | 57.3 | del(13q) | Mutated | Pos |
| 2 | 633 | 33.8 | Tri12 | Mutated | Unk |
| 3 | 941 | 104.8 | Neg | Unmutated | Pos |
| 4 | 181 | 52.4 | del(13q) | Mutated | Neg |
| 5 | 494 | 71.5 | del(13q) | Unmutated | Neg |
| 6 | 777 | 94.2 | del(13q) | Mutated | Unk |
| 7 | 425 | 138.9 | del(13q) | Unmutated | Pos |
| 8 | 210 | 55.2 | del(17p) | Unmutated | Pos |
| 9 | 683 | 60.7 | tri12, del(17p) | Unmutated | Neg |
| 10 | 536 | 20.1 | Neg | Mutated | Neg |
| 11 | 357 | 120.5 | del(13q) | Mutated | Unk |
| 12 | 801 | 18.4 | tri12 | Mutated | Unk |
| 13 | 848 | 80.8 | del(13q) | Mutated | Unk |
| 14 | 279 | 38.3 | Neg | Mutated | Unk |
| 15 | 378 | 21.1 | del(11q), del(17p) | Mutated | Unk |
| 16 | 375 | 21.7 | del(13q) | Mutated | Unk |
| 17 | 540 | 25.3 | del(17p) | Mutated | Neg |
| 18 | 445 | 48.7 | Neg | Mutated | Unk |
| 19 | 592 | 71.7 | del(13q) | Mutated | Neg |

FISH, fluorescence in situ hybridization; del(17p), deletion of 17p; del(13q), deletion of 13q; del(11q), deletion of 11q; tri12, chromosome 12 trisomy; pos, positive; neg, negative, unk, unknown.

**Supplemental Table 2. List of antibodies.**

| **Protein** | **kDa** | **Antibody name** | **Dilution** | **Supplier** | **Catalog No.** |
| --- | --- | --- | --- | --- | --- |
| Vinculin | 124 | Vinculin (E1E9V) XP® Rabbit | 1:1000 | Cell Signaling | 13901 |
|  |  |  |  |  |  |
| PARP | 113 | PARP antibody [GT6212] | 1:1000 | GeneTex | GTX628836 |
| P62 | 62 | p62 (human) polyclonal antibody | 1:2000 | Enzo | BML-PW9860-0025 |
| GAC/KGA | 58 | KGA/GAC Polyclonal antibody | 1:1000 | Proteintech Group | 12855-1-AP |
| β-actin | 42 | beta Actin antibody [GT5512] | 1:1000 | GeneTex | GTX629630 |
| MCL1 | 40 | Mcl-1 (D2W9E) Rabbit Ab | 1:1000 | Cell Signaling | 94296 |
| BCL-XL | 30 | Bcl-xL (54H6) Rabbit mAb | 1:1000 | Cell Signaling | 2764S |
| LC3A/B | 14,16 | LC3A/B Antibody | 1:1000 | Cell Signaling | 12741 |
| BCL2 | 26 | Bcl-2 (124) Mouse Ab | 1:1000 | Cell Signaling | 15071S |
|  |  |  |  |  |  |

**Supplementary Table 3. Combination index values of drug combinations.**

| **Cell line** | **Drug combination** | **Combination index value*** | | | | **Comments** |
| --- | --- | --- | --- | --- | --- | --- |
|  |  | **ED50** | **ED75** | **ED90** | **ED95** |  |
| **HG-3** | Venetoclax + CB-839 | 0.35 | 0.4 | 1.06 | 2.3 | **Additive effect** |
|  | Ibrutinib + CB-839 | 0.10 | 0.32 | 1.02 | 2.25 | **Additive effect** |
|  | AZD-5991 + CB-839 | 0.19 | 0.05 | 0.02 | 0.02 | **Synergism** |
| **MEC-1** | Venetoclax + CB-839 | 0.46 | 0.51 | 0.58 | 0.63 | **Synergism** |
|  | Ibrutinib + CB-839 | 2.57 | 0.29 | 0.03 | 0.007 | **Additive effect** |
|  | AZD-5991 + CB-839 | 0.1 | 0.12 | 0.16 | 0.19 | **Synergism** |

***A combination index value of 1 represents an additive effect; a value greater than 1 represents an antagonistic effect; and a value less than 1 represents a synergistic effect.**

*(ombination index (CI) 1 or >1 or <1 represent additive, antagonistic and synergistic)

**Supplemental Figure 1. Gating strategy to evaluate ROS levels**

**
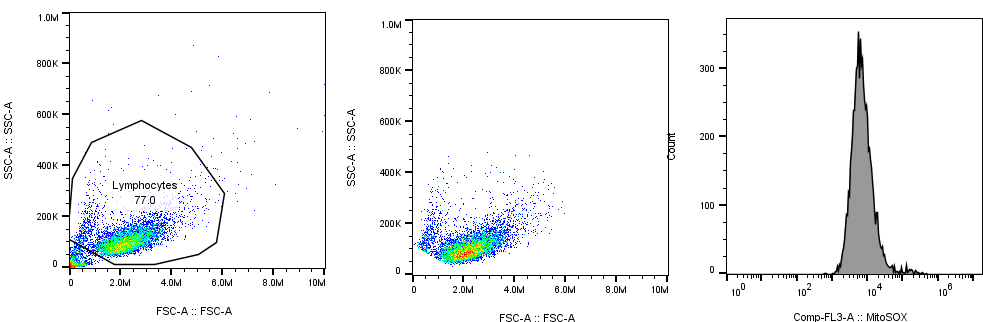
**

**
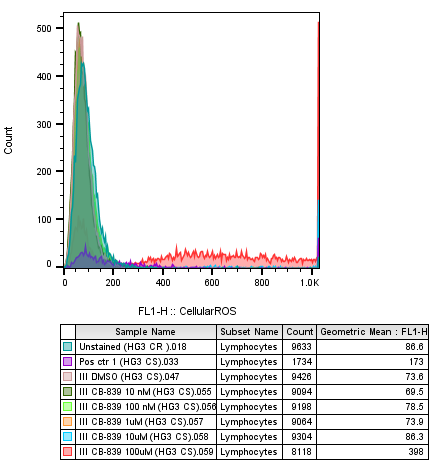

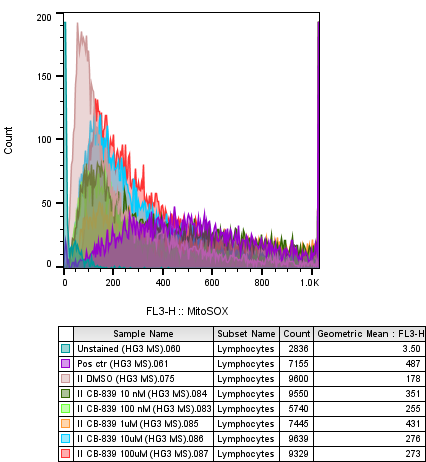
**

Total of 10,000 events were acquired, and the population of lymphocytes was gated on the forward scatter (FSC) versus side scatter (SSC) plot. Histograms for FL-1 and FL-3 channels were generated to evaluate fluorescence intensity for mitochondrial SOX (MitoSOX) and cellular ROS, respectively. Further, the histograms were layered on a single plot, and the geometric mean value was used to evaluate ROS production. Treatment groups were compared using an unpaired, two-tailed Student's t-test to the DMSO group. FCCP 5 μM served as a positive control for mitochondrial SOX, and tert-butyl hydroperoxide 5 μM served as a positive control for cellular ROS. DMSO, dimethyl sulfoxide; FCCP, carbonyl cyanide-p-trifluoromethoxyphenylhydrazone.

**Supplemental Figure 2**. **Inhibition of cellular glutathione levels and changes in superoxide and reactive oxygen species levels after treatment with** **L-buthionine-(S,R)-sulfoximine.
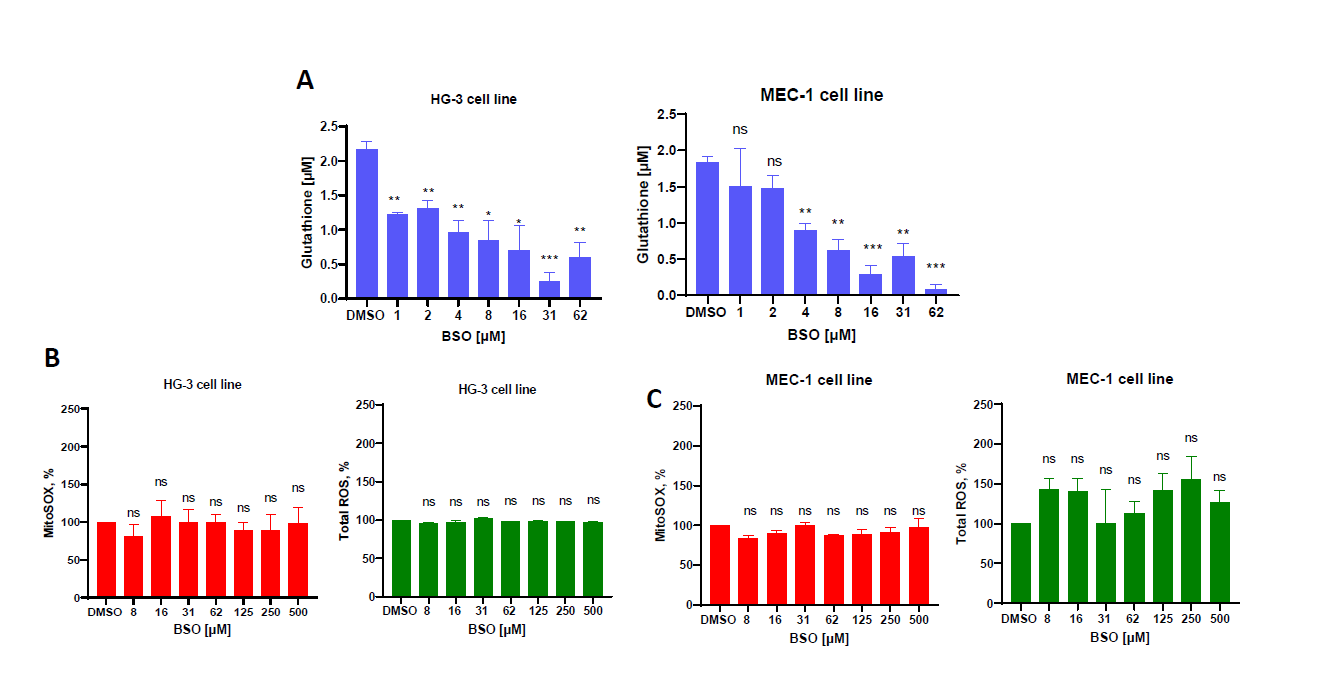
**

(**A**) A decline in glutathione levels was seen upon inhibition of gamma-glutamyl-cysteine synthetase. Cells were treated with L-buthionine-(S,R)-sulfoximine (BSO; 1-62 μM) for 72 hours, and the levels of glutathione were determined using assay kits as described in the Methods section. Changes in (**B**) the mitochondrial superoxide (mitoSOX) levels and (C) the total cellular reactive oxygen species (ROS) levels were evaluated in HG3 and MEC-1 cells. The ROS levels were measured using flow cytometry after 4 hours of treatment per the manufacturer’s protocol. The mitoSOX levels were measured after 24 hours of incubation. Data represent the mean ± the standard error of the mean for 3 biologically separate experiments. DMSO, dimethyl sulfoxide; ns, not significant. **P* < 0.05; ***P* < 0.01; ****P* < 0.001.

**Supplemental Figure 3.** **Protein expression changes after CB-839 treatment.**

**
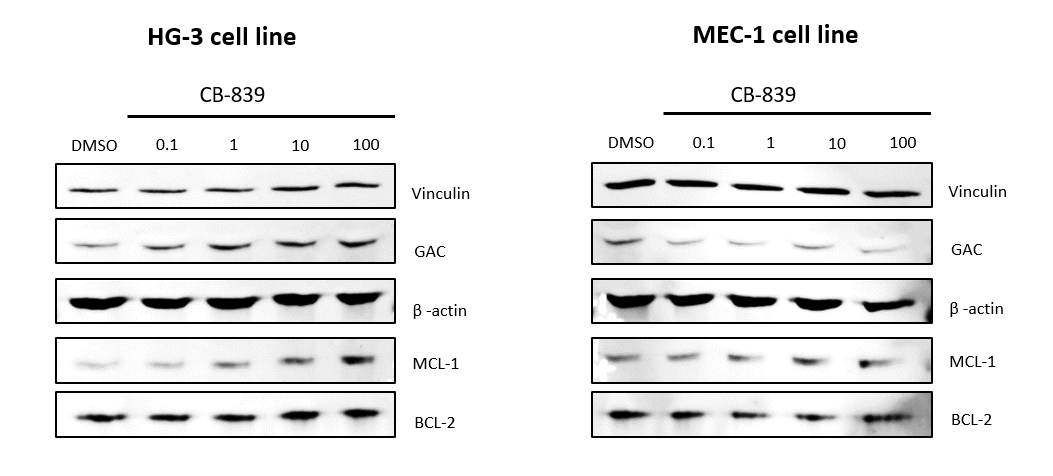
**

Glutaminase C (GAC), myeloid cell leukemia-1 (MCL-1), and B-cell lymphoma-2 (BCL-2) protein expression after 72 hours of incubation with CB-839 (0.01-100 μM) in the HG3 and MEC-1 cell lines. DMSO, dimethyl sulfoxide.

**Supplemental Figure 4.** **Changes in radioactive thymidine and uridine incorporation upon CB-839 treatment.**


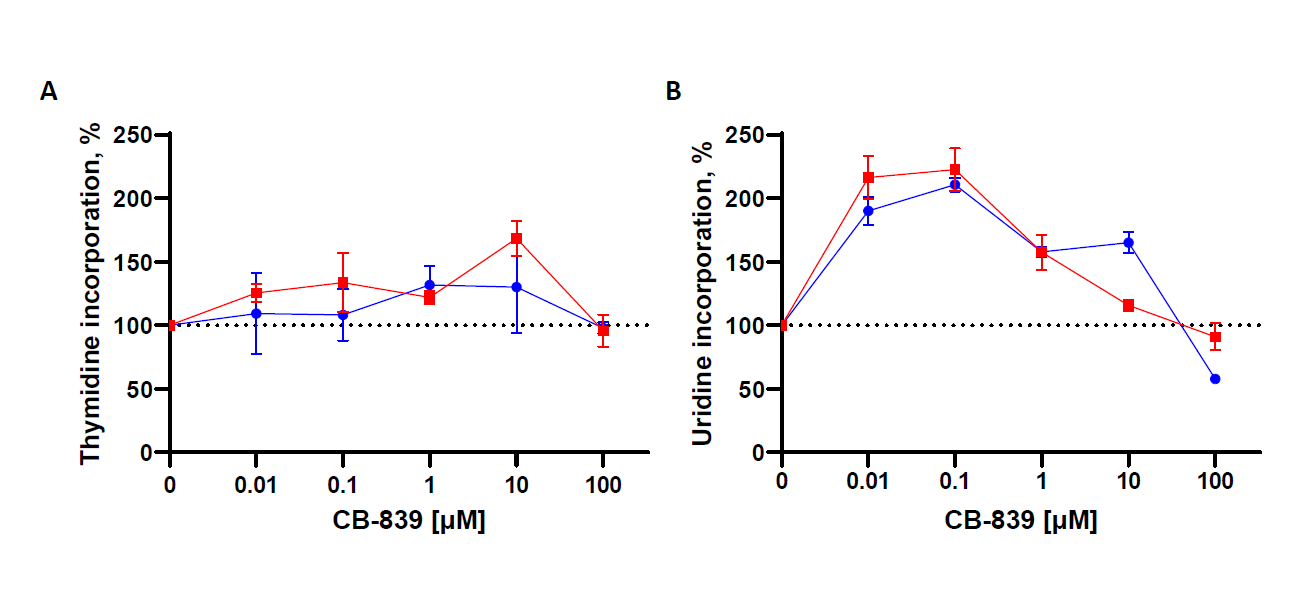


After 72 hours of incubation with CB-839 (0.01-100 μM), the incorporation of exogenous radioactive (**A**) thymidine and (**B**) uridine was assessed, and compensatory DNA and RNA synthesis increases were detected. Data represent the mean ± the standard error of the mean for 3 biologically separate experiments. Red lines represent HG-3 cells while blue lines represent MEC-1 cells.

**Supplemental Figure 5. The autophagy inhibitor chloroquine did not induce apoptosis in CB-839-treated cells.**

**
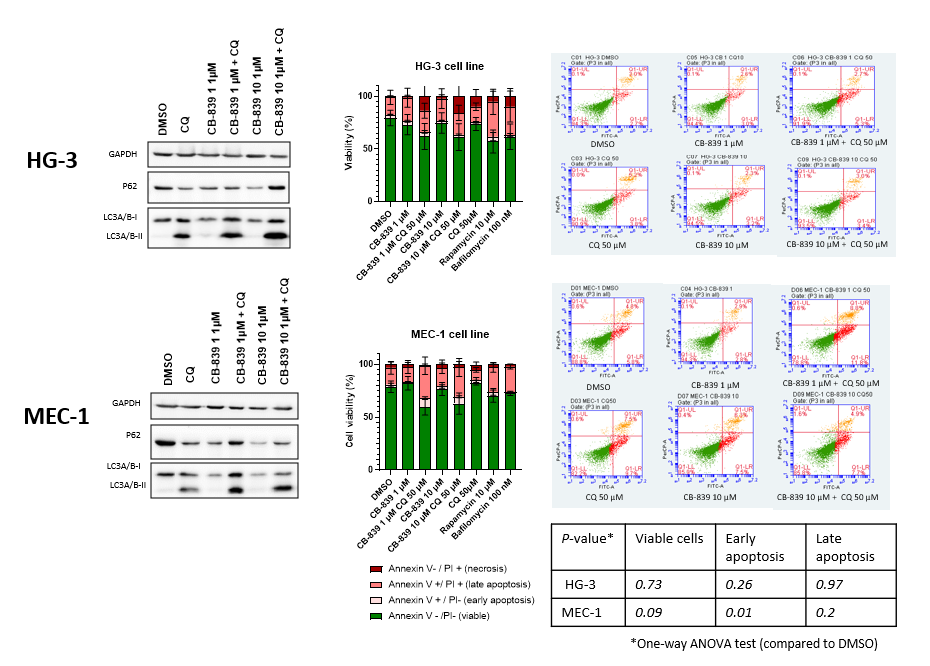
**

HG-3 and MEC-1 cells were incubated with CB-839 (1 μM or 10 μM) for 24 hours, with or without chloroquine (CQ; 50 μM). The autophagy inducer rapamycin (10 μM) and the autophagy inhibitor bafilomycin A1 (100 nM were used as positive and negative controls, respectively. The apoptosis rate was measured using annexin V/propidium iodide (PI) staining, and the percentages of viable (green), early apoptotic (pink), late apoptotic (red), and necrotic cells (dark red) were evaluated. A 1-way analysis of variance test was performed in the first 3 groups compared to the dimethyl sulfoxide (DMSO) control. *P* values are represented in the table. Data are shown as the mean ± the standard error of the mean. GAPDH, glyceraldehyde 3-phosphate dehydrogenase; LC3, light chain 3.

**Supplemental Figure 6. The results of Seahorse mitochondrial stress testing in chronic lymphocytic leukemia samples from 5 patients.**


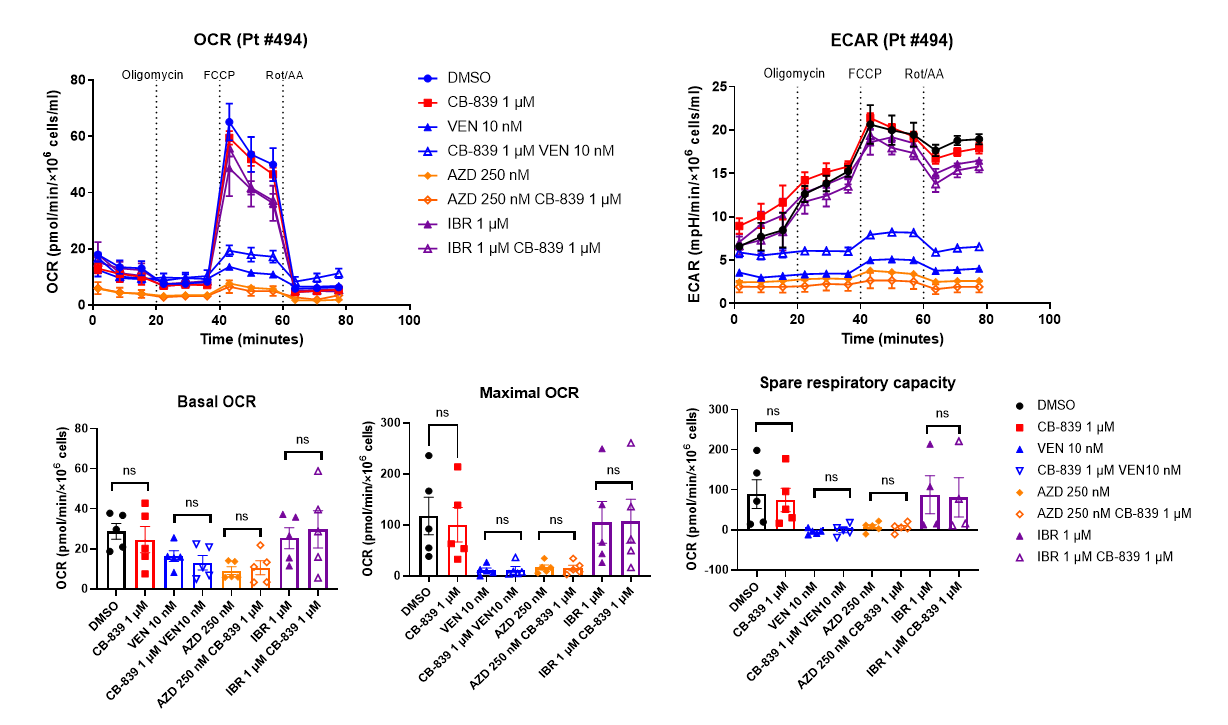


Oxygen consumption rate (OCR) and extracellular acidification rate (ECAR) curves for individual chronic lymphocytic leukemia (CLL) patients’ peripheral blood samples. The summary graphs show the basal OCR, maximal OCR, and spare respiratory capacity. Data are presented as mean ± the standard error of the mean. There was a minimum of 3 replicates for each sample and condition. AZD, AZD-5991; DMSO, dimethyl sulfoxide; FCCP, carbonyl cyanide-p-trifluoromethoxyphenylhydrazone; IBR, ibrutinib; ns, not significant; Rot/AA, rotenone/antimycin A; VEN, venetoclax.

**Supplemental Figure 7. Nucleoside triphosphate pool changes induced by glutaminase enzyme inhibition.**


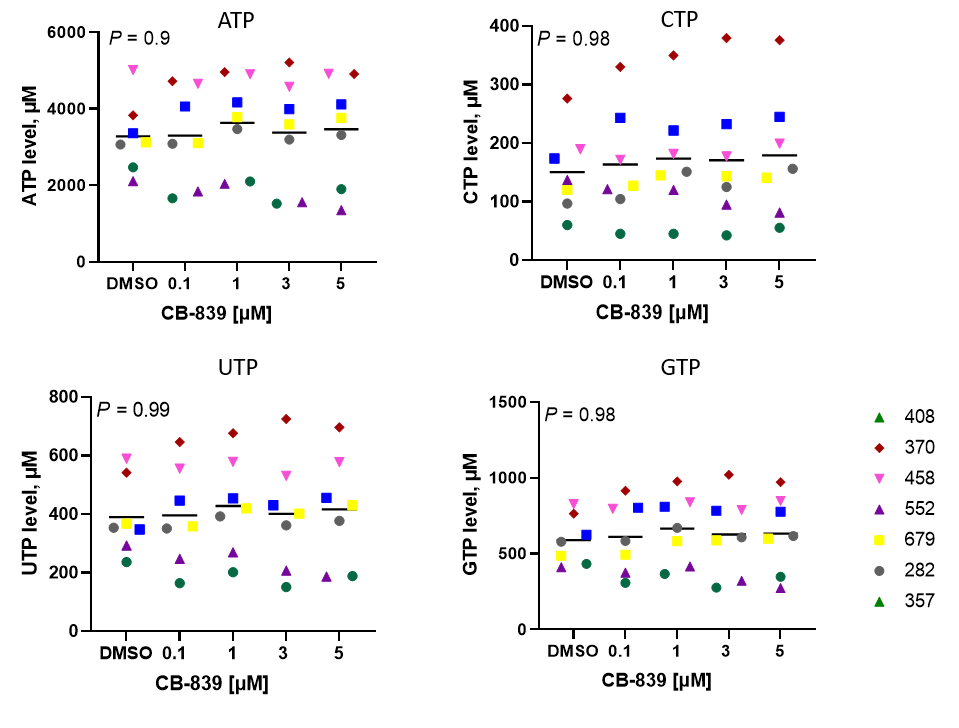


High-performance liquid chromatography was used to assess the nucleoside triphosphate (adenosine triphosphate [ATP], cytidine triphosphate [CTP], uridine triphosphate [UTP], and guanosine triphosphate [GTP]) concentration changes in primary chronic lymphocytic leukemia lymphocytes (n = 7) after 8 hours of incubation with CB-839. The data are presented as the mean ± the standard error of the mean. The significance was evaluated using a 1-way analysis of variance test. DMSO, dimethyl sulfoxide.

**Supplemental Figure 8. Comparison of IC_50_ values for monotherapy versus CB-839 combination treatment.**

| **Cell line** | CB-839 | Venetoclax | CB-839 + venetoclax | Ibrutinib | CB-839 + ibrutinib | AZD-5991 | CB-839  + AZD-5991 |
| --- | --- | --- | --- | --- | --- | --- | --- |
| MEC-1 | 40 μM | 2.9 μM | 0.61 μM | 1.2 μM | 0.89 μM | 1.9 μM | 0.38 μM |
| HG3 | 4.4 μM | 8.1 μM | 1.4 μM | 1.3 μM | 0.84 μM | 9.9 μM | 0.5 μM |

IC_50_ values were calculated using an MTS assay and CompuSyn software analysis. The growth inhibitory curves compare CB-839, venetoclax, AZD-5991, and ibrutinib alone and in combination. Data are shown as the mean ± the standard error of the mean.


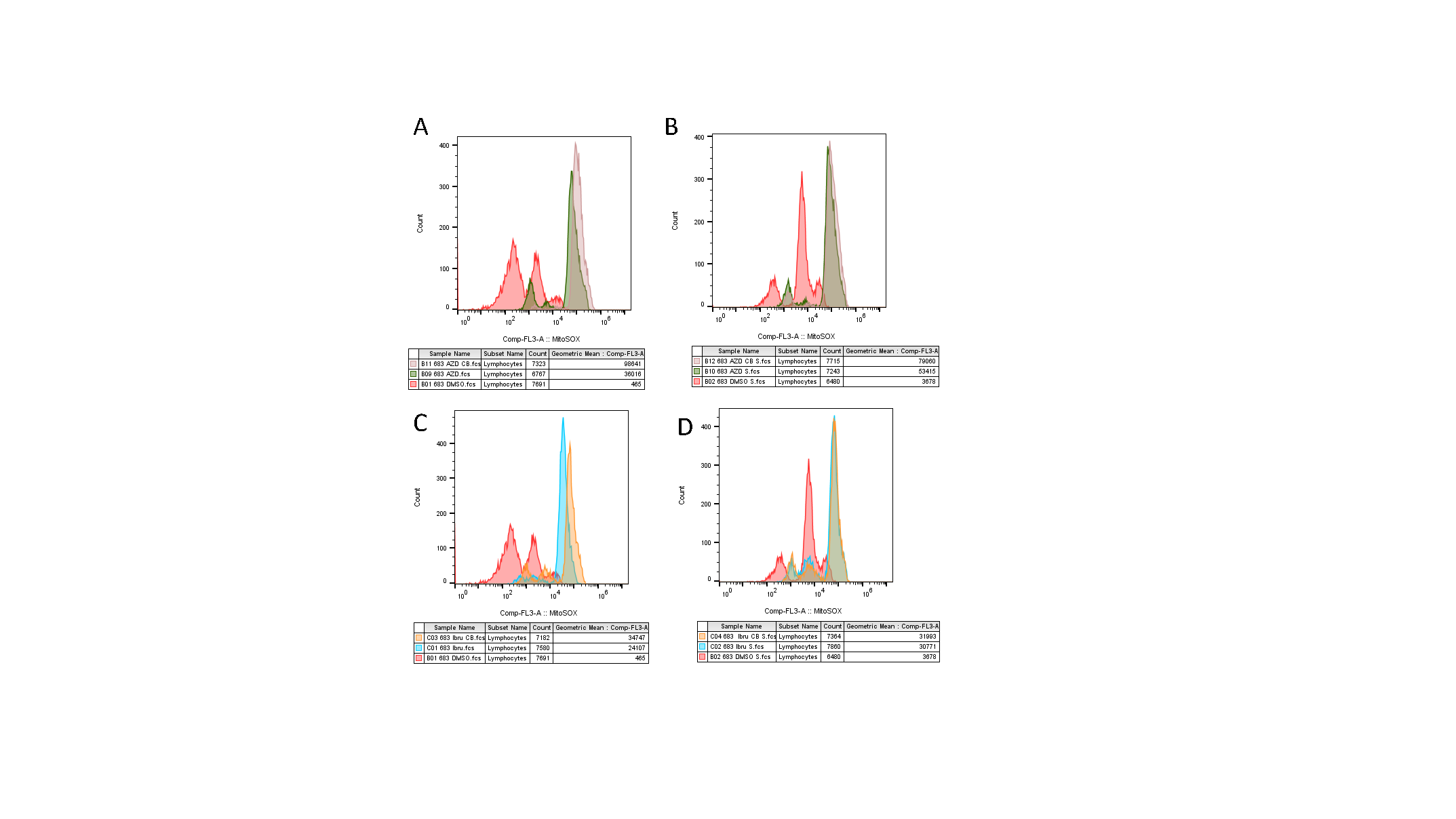
**Supplemental Figure 9. Mitochondrial superoxide production in a patient with tri12 and del(17p) after 72 hours of treatment.**

A single case of abnormally high mitochondrial superoxide (mitoSOX) production. The patient had chromosome 12 trisomy and del(17p). The graphs show the effects of incubating the cells with (**A, B**) AZD-5991 (AZD) and (**C, D**) ibrutinib (Ibru), with and without the addition of CB-839. Cells with (**A, C**) no cytokine stimulation and (**B, D**) CD40 ligand and interleukin-4supplementation are represented. DMSO, dimethyl sulfoxide.
